# Ultrasound imaging of bone fractures

**DOI:** 10.1186/s13244-022-01335-z

**Published:** 2022-12-13

**Authors:** Giulio Cocco, Vincenzo Ricci, Michela Villani, Andrea Delli Pizzi, Jacopo Izzi, Marco Mastandrea, Andrea Boccatonda, Ondřej Naňka, Antonio Corvino, Massimo Caulo, Jacopo Vecchiet

**Affiliations:** 1 Unit of Ultrasound in Internal Medicine, Department of Medicine and Science of Aging, G. D’Annunzio University, Chieti, Italy; 2 Physical and Rehabilitation Medicine Unit, Luigi Sacco University Hospital, ASST Fatebenefratelli-Sacco, Milan, Italy; 3 Unit of Radiology, “Santissima Annunziata” Hospital, Chieti, Italy; 4 Department of Innovative Technologies in Medicine and Dentistry, G. D’Annunzio University, Chieti, Italy; 5 Internal Medicine, Bentivoglio Hospital, AUSL Bologna, Bologna, Italy; 6 First Faculty of Medicine, Institute of Anatomy, Charles University, Prague, Czech Republic; 7 Motor Science and Wellness Department, Parthenope University, Naples, Italy; 8 Department of Neuroscience Imaging and Clinical Sciences, G. D’Annunzio University, Chieti, Italy; 9 Clinic of Infectious Diseases, Department of Medicine and Science of Aging, G. D’Annunzio University, Chieti, Italy

**Keywords:** Ultrasound, Bone fracture, Fracture healing

## Abstract

Ultrasound imaging is widely used to evaluate the neuromusculoskeletal system, and recently, a particular interest is mounting in assessing the bone tissue and fractures. Ultrasound can be considered a valuable diagnostic tool to perform a first-line evaluation of bone tissue, especially in particular settings without direct access to X-ray imaging and/or in emergency conditions. Moreover, different healing phases of bone fractures can be accurately assessed by combining the B-mode modality and (high-sensitive) color/power Doppler optimizing the management of patients—e.g., planning of progressive loads and rehabilitation procedures. In this review, we summarized the role of ultrasound imaging in the management of bone fractures and described the most common sonographic signs encountered in the daily practice by assessing different types of bone fractures and the progressive phases of the healing process.

**Supplementary Information:**

The online version contains supplementary material available at 10.1186/s13244-022-01335-z.

## Key points

- US represents an alternative to XR for the occult bone fractures diagnosis.
- Knowledge of sonographic patterns is crucial for the detection of challenging fractures.
- US allows an early assessment of the callus formation and bone healing.

**Supplementary Information:**

The online version contains supplementary material available at 10.1186/s13244-022-01335-z.

## Introduction

Ultrasound (US) imaging is a diagnostic technique characterized by several advantages; indeed, it is a cost-effective, non-invasive, reproducible examination and, unlike X-ray (XR), does not use ionizing radiation, resulting in a safe technique, particularly in the pediatric population [1, 2]. In children, US allows studying the cartilaginous components of immature bones, which are poorly evaluated on XR, and still represents an important diagnostic challenge [3]. For instance, in non-displaced torus forearm fractures (i.e., Buckle fractures) and “greenstick” fractures, it has been shown that US is comparable to XR for both diagnosis and management [4, 5]; moreover, several authors have demonstrated the pivotal role of US in the diagnosis of costal cartilage injuries in the pediatric population [2, 6, 7]. US is already widely used in musculoskeletal imaging for the assessment of superficial soft tissue illnesses [8, 9], articular and periarticular pathologies [10], muscle disorders [11, 12], nerve injuries [13], and tendinopathies [14].

Recently, particular interest has growth in the diagnostic potentialities of US in bone tissue pathologies [15]. Some authors have proposed the role of US as an alternative tool to conventional XR in the diagnosis of pediatric fractures and occult fractures in the adult population [1, 16]. The latter are usually overlooked on conventional XR, especially in patients with foot and ankle trauma, [1, 16–18] and in cases of rib fractures. Interestingly, in the pertinent literature, chest ultrasonography has shown a sensitivity of 89.3% and a specificity of 98.4% compared to computed tomography (CT) imaging for the diagnosis of any rib fracture [19]. The sonographic examination represents a potential alternative to XR for the diagnosis of scaphoid and metatarsal stress fractures; moreover, it allows early identification of Hill–Sachs lesions which are often undetectable on XR and require other imaging modalities like CT and magnetic resonance imaging (MRI) [18].

In the emergency setting, US imaging can be performed to assess in real-time the correct reduction in distal radius fractures [20, 21] and to support the diagnosis of fractures of the long bones in adult patients—hemodynamically unstable—during resuscitation phases [22, 23]. US can be performed also to monitor the callus formation [15] identifying it earlier than conventional XR in which it is visualized starting from 10 weeks after trauma. In this way, US can be used in the follow-up of fracture healing to promptly diagnoses an eventual delayed union of the bony fragments [15].

In this pictorial review, we summarize the main imaging features on US assessment of bone fractures. Starting from the normal sonographic appearance of the bone and superficial soft tissues, here we describe pathological findings of different types of bone lesions and the sonographic patterns of different healing phases.

## Sono-anatomy of bone and related soft tissues

Imaging of the bone is traditionally performed with standard XR, CT, MRI, or scintigraphy. The first-line imaging tool in fracture diagnosis is represented by standard X-ray, while US is traditionally used for the evaluation of surrounding soft tissue [15, 24]. Likewise, it has been widely demonstrated that US imaging can demonstrate occult fractures often undetected by the previous X-ray examination [25–28].

**Fig. 1:**
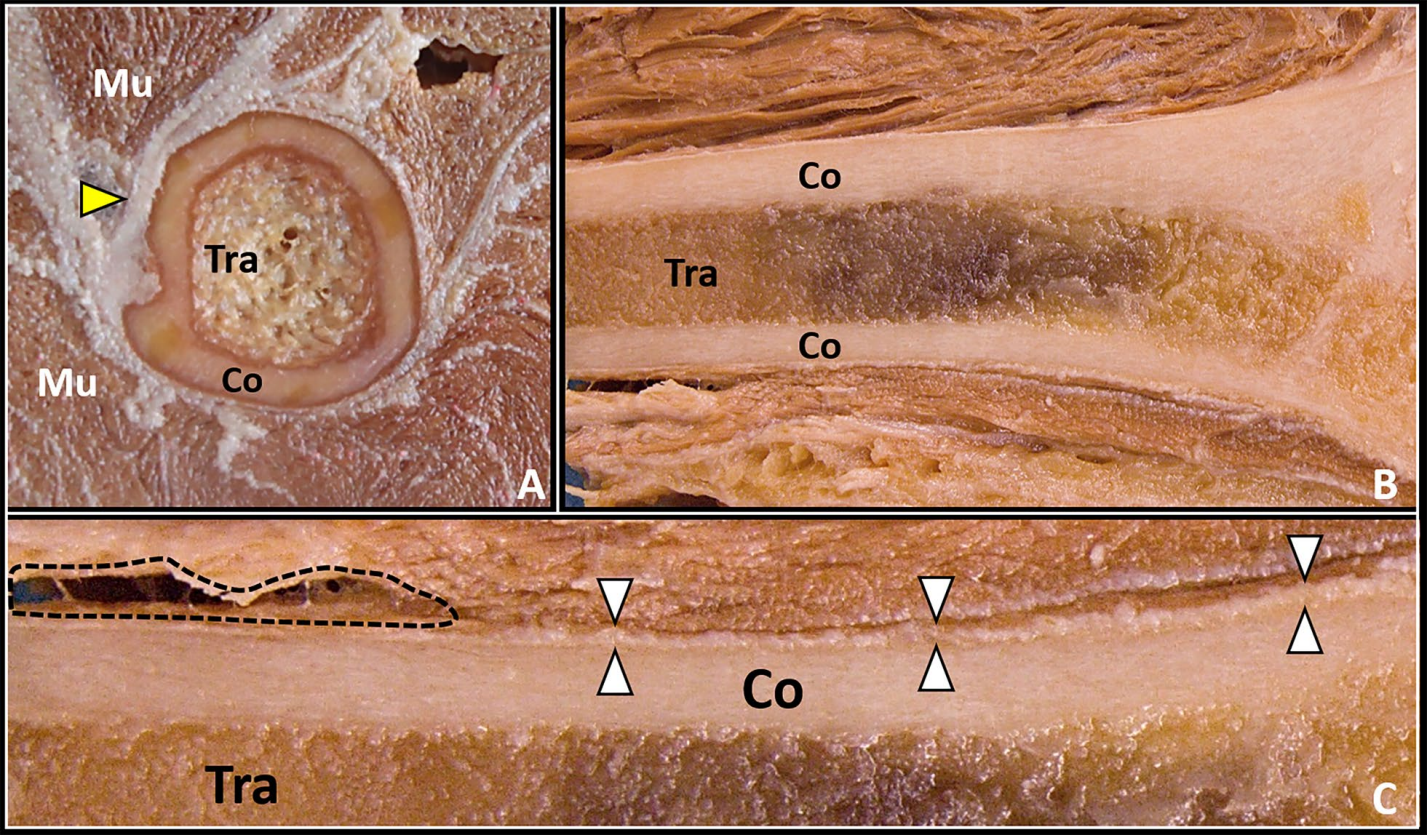
Cadaveric anatomy of bone tissue. The bone presents an inner portion with a trabecular texture (*Tra*) and an outer component—compact in nature—known as cortical bone (*Co*) (**A**, **B**). Of note, the periosteum (*white arrowheads*) tightly envelops the surface of the bone and, if damaged, allows the blood to diffuse toward the epi-periosteal space (*black dotted line*) (**C**). Mu: muscle tissue, yellow arrowhead: fat tissue

Sonographic assessment of the superficial bones must be performed with high-frequency transducers—usually more than 8 MHz—that guarantee high spatial resolution but limited penetration in depth [29]. For deeper bones (e.g., the femur), lower frequencies (2–5 MHz) are used, which have a less spatial resolution but higher penetration capacity [29]. Multiple scan planes and acoustic windows are usually coupled to acquire a panoramic view of the fracture line and of the spatial localization of bony fragments [30]. Ultrasound evaluation of the bone tissue is mainly based on the different acoustic impedance between the cortical bone and the surrounding soft tissues [31]. Considering its histological architecture only the bone surface can be sonographically visualized while the inner portion of the bone—i.e., the trabecular bone is not evaluable (Fig. 1) [15, 32].

**Fig. 2:**
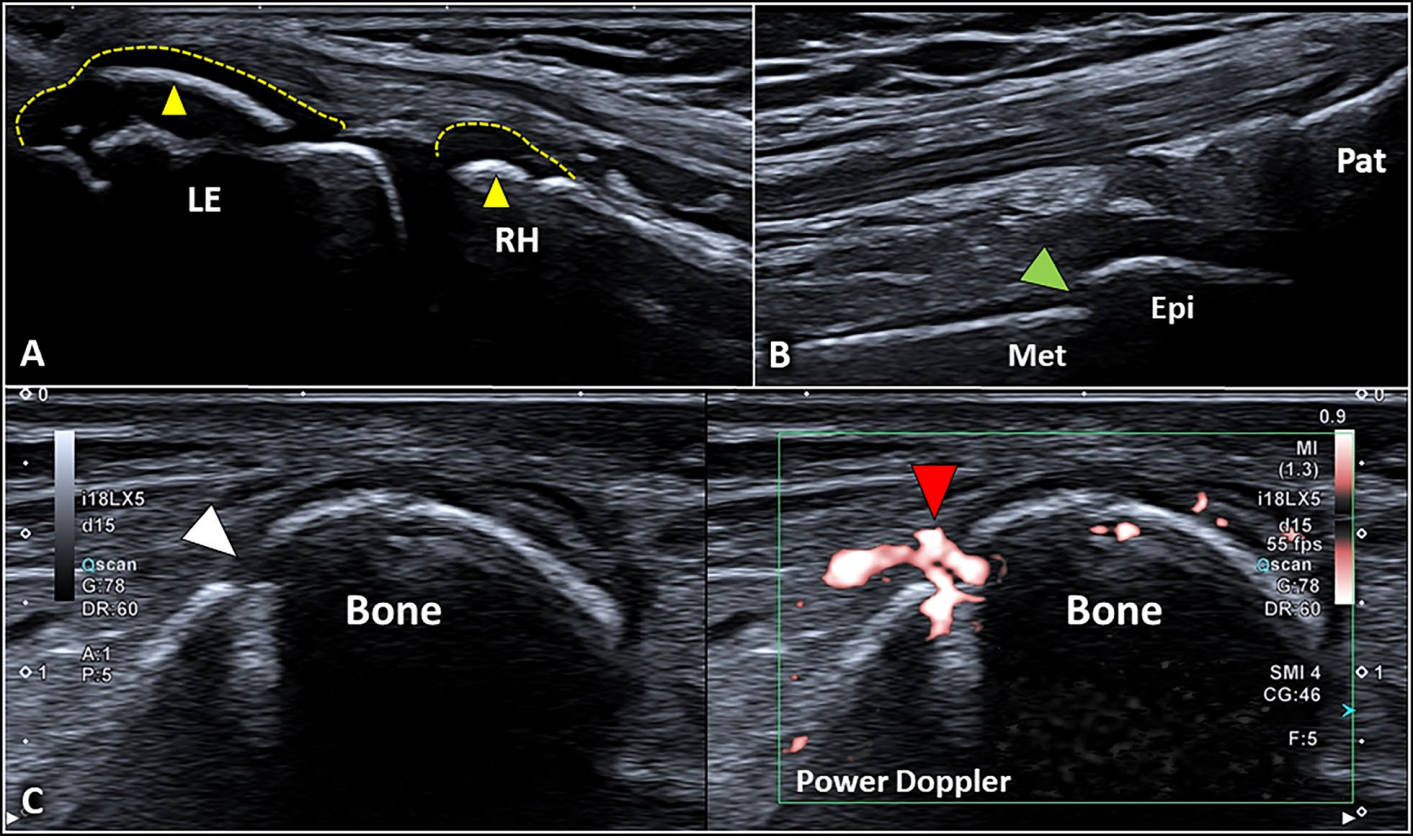
Normal sonographic findings of the bone tissue. A longitudinal view of the lateral elbow in a young volunteer clearly shows the cartilaginous epiphysis (*yellow dotted lines*) of the radial head (*RH*) and lateral epicondyle (*LE*); of note, the hyperechoic lines (*yellow arrowheads*) within the hyaline cartilage are the epiphyseal ossification centers (**A**). Likewise, a longitudinal scan of the suprapatellar region in the knee shows the physis (*green arrowhead*) in between the metaphysis (*Met*) and epiphysis (*Epi*) of the distal femur in a child (**B**). Importantly, focal interruption of the (hyperechoic) cortical bone can be related to the presence of nutritional foramina (*white arrowhead*) crossed by feeding vessels (*red arrowhead*) (**C**). *Pat* patella

Comprehensive knowledge of the sonographic patterns of different anatomical structures of the neuromusculoskeletal system in physiological conditions is paramount to correctly interpreting the ultrasound findings in pathological conditions. The bone surface presents as a hyperechoic line [15]—with the reverberation artifact [31] visible in-depth with respect to the cortical bone—wrapped by the periosteal lining. The periosteum, in the adults, appears as a scarcely visible hypoechoic band, while in the children population it is thicker and more easily visible with ultrasound [33, 34]. We strongly suggest to always evaluate also the superficial soft tissues surrounding the bone segments, where often several indirect sonographic signs of bone lesions can be easily identified. The dermo-epidermal complex appears as a trilaminar structure with a superficial hyperechoic line representing the epidermis, an intermediate hypoechoic thin band representing the dermis, and a deep hyperechoic line representing the dermo-hypodermal interface [8, 9]. The subcutaneous tissue (hypodermis) shows hypoechoic fat lobules stabilized by hyperechoic septa representing the fibrous scaffold of the subcutis [8, 9]. Of note, within the connective scaffold, the lymphovascular branches are located. The hypodermis is separated from the underlying muscles by the deep fascia—a multi-layered hyperechoic band [11]. In order to perform a rigorous sonographic examination, the knowledge of several physiological condition potentially mimicking bone fractures is essential. Nutritional vessels penetrating the bone cortex (Additional file 2: Video 1), (cartilaginous) growth plates in between the metaphysis and epiphysis in children’s population (Additional file 3: Video 2), [35, 36] accessory ossification centers—appearing as bone fragments with a rounded edge and separated from the adjacent main bone, and attachment zones of tendons and ligaments are the most common examples of physiological bone discontinuities (Fig. 2).

## Pathological sonographic findings in bone fractures

Due to the high density and compact structure of cortical bone, US waves are reflected determining a hyperechoic (bright) line which represents the cortical bone surface [31]. Bone fractures can be sonographically visualized as a cortical contour interruption [15, 28]. US imaging also allows the identification of surrounding hematoma and the evaluation of bone callus during its different phases [32]. Indeed, the callus presents as an anechoic/hypoechoic formation in the initial healing phases allowing the ultrasound to pass through. Over time, it progressively evolves into hyperechoic bone tissue, completely reflecting the US beam [15, 31]. By definition, a fracture is a bone discontinuity caused by a mechanical force exceeding the bone’s withstanding ability. Among various classifications, bone fractures can be divided into impact fractures due to direct trauma, avulsion fractures due to tractional forces, and stress fractures [28]. From a clinical point of view, not only the sonographic patterns of bone and related soft tissues but also the medical history and physical examination should be always considered in order to accurately classify the bone lesions in daily practice. Below, we have reported the most common sonographic signs for the aforementioned three main types of bone fractures. Of note, different types of bone lesions can often coexist simultaneously in the same patient—e.g., in patients with high-energy complex trauma.

## Impact fractures

Impact fractures can occur in individuals of all ages, and their shape and anatomical location are highly dependent on several factors—e.g., the individual bone quality and the dynamics of trauma. For instance, in elderly people, impact fracture may also occur after a minor trauma due to pathological conditions affecting the resistance of bone tissue such as osteoporosis and vitamin D deficiency [37]. Ultrasound assessment of impact fractures is technically easier for diaphysis and metaphysis of long bones considering the linear shape of the cortical bone; instead, it is more challenging for small and irregularly shaped bones as in the wrist and foot [28]. In the latter cases, an optimal position of the patient/anatomical segment is paramount to accurately expose as much surface as possible of the cortical bone, and multiple acoustic windows of the target bone should be coupled to acquire a panoramic view of the fracture line and bony fragments [30].

Several authors have demonstrated that the diagnostic accuracy of ultrasound imaging in identifying impact fractures is higher at the level of diaphysis of long bones, and lesser at the ends-of-bones and near the joints [38]. Likewise, in the pertinent literature, sonographic evaluation of bone fractures not involving joints can be comparable to radiography with a sensitivity of 0.94 and specificity of 0.92 [39].

**Fig. 3:**
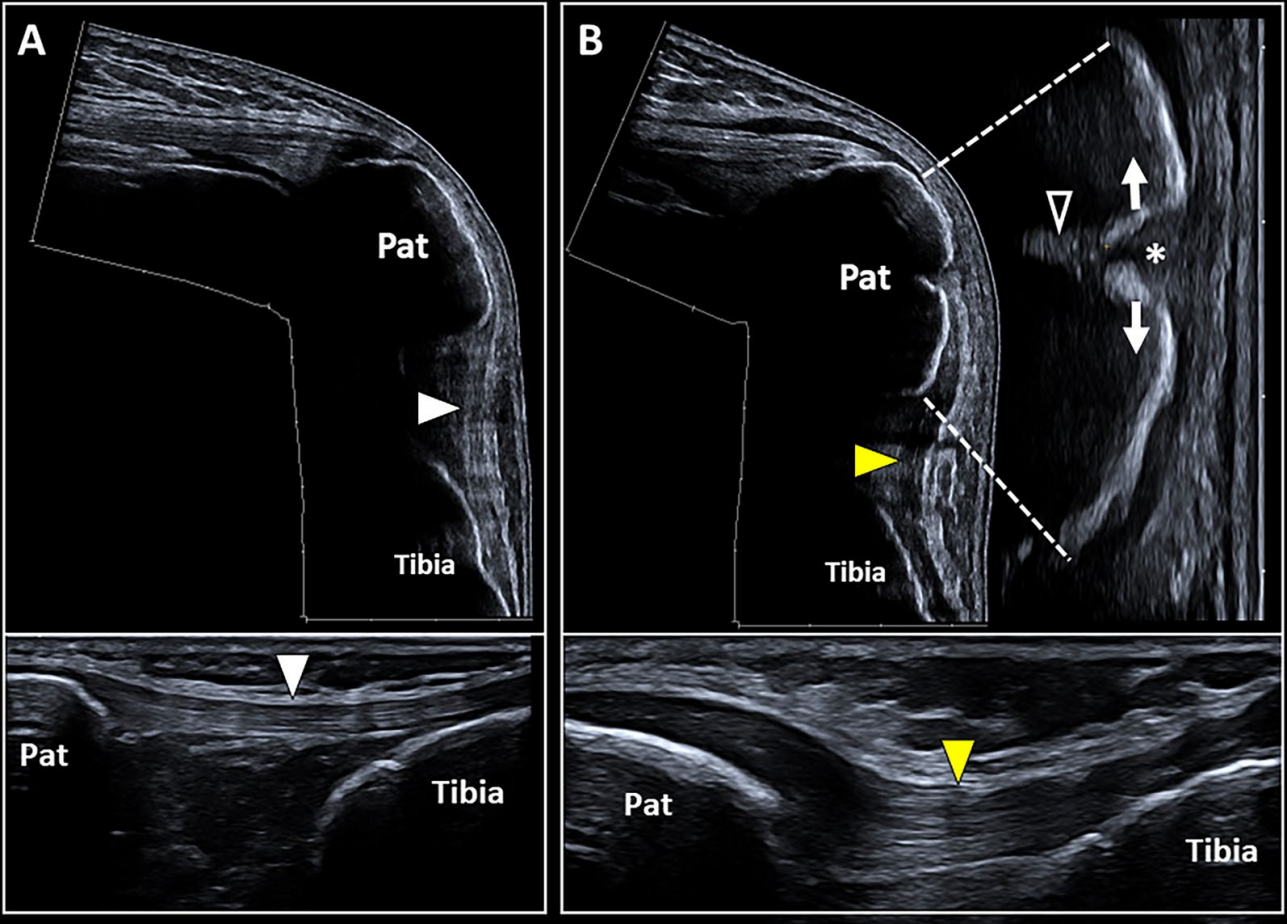
Pathological sonographic findings of the bone tissue. The comparative sonographic assessment shows a continued hyperechoic cortical bone of the patella (*Pat*) with a tensioned patellar ligament (*white arrowhead*) on the healthy side (**A**); instead, cortical defect (*white asterisk*) of the patella (*Pat*), diastasis of bony fragments (*white arrows*), and deformation of the patellar ligament (*yellow arrowhead*) are clearly visible in the post-traumatic knee (**B**). Of note, the disruption of the bony cortex allows the US beam to partially penetrate within the bone tissue generating an echoic wedge (*void arrowhead*) (**B**)

**Fig. 4:**
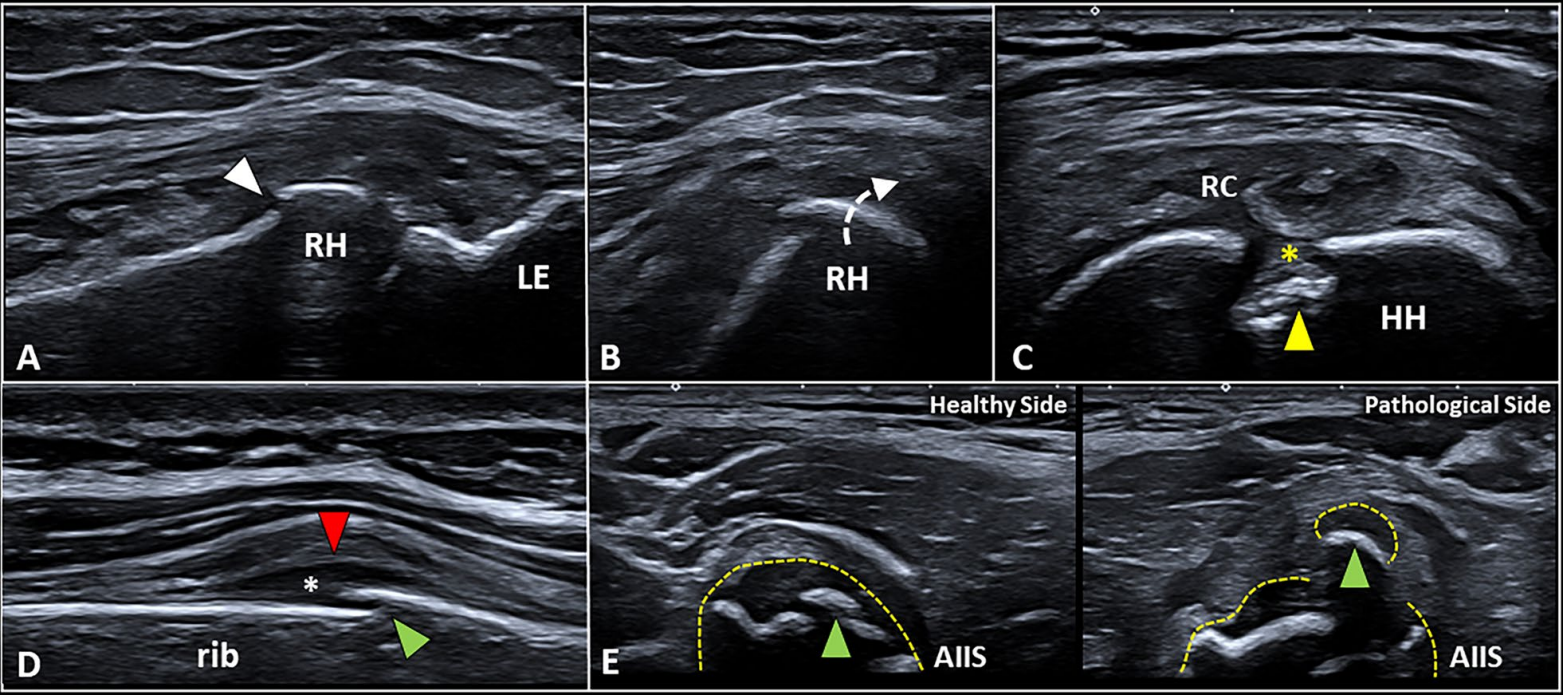
Impact fractures and avulsion fractures. Longitudinal view (**A**) shows the impact fracture (*white arrowhead*) of the radial head (*RH*), but only by performing the transverse scan (**B**) the degree of rotation (*white dotted arrow*) of the bony fragment can be clearly observed. Likewise, cortical bone depression (*yellow arrowhead*) on the posterior surface of the humeral head (*HH*)*—*filled with fibrotic tissue (*yellow asterisk*)*—*can be observed in a patient with previous anterior subluxation of the shoulder (**C**). Unlike the post-acute injuries, in the acute phase of trauma (**D**) the misalignment of the cortical bone (*green arrowhead*) is usually coupled with the periosteal bulging (*red arrowhead*) and subperiosteal hematoma (*white asterisk*). Of note, avulsion fractures in the pediatric population (**E**) can show a simultaneous shifting of the cartilaginous epiphysis (*yellow dotted line*) and the epiphyseal ossification center (*green arrowhead*) located within the hyaline cartilage. *LE* lateral epicondyle, *RC* rotator cuff, *AIIS* anterior inferior iliac spine

Longitudinal (Additional file 4: Video 3) and transverse (Additional file 5: Video 4) scans should be coupled in each and every patient to confirm the focal interruption of the hyperechoic cortical bone. Indeed, the longitudinal scan allows a panoramic view of the cortical irregularity (Fig. 3) while the transverse scan often guarantees a better visualization of the shift/rotation of the pathological bony fragment. The proximal humerus (Additional file 6: Video 5), and ribs, is usually considered the anatomical segments where impact fractures are more frequently identified during the ultrasound imaging in daily practice (Fig. 4) [28, 40]. Interestingly, standard radiography may not reveal non-displaced fractures of the humeral head (e.g., Hill–Sachs fracture) or require particular projections to visualize them. Of note, not only impact fractures of the shoulder involving the greater tuberosity of the humeral head but also fractures with atypical anatomical location—e.g., the body of scapula and coracoid process—can be sonographically detected in patients with persistent shoulder pain after an acute trauma [28].

We strongly suggest accurately evaluating not only the focal interruption of the cortical bone but also the shape/spatial arrangement of the hematoma located around the bone fracture because it can be considered an indirect sonographic sign of the periosteal integrity [2]. Indeed, in fractures with anatomical preservation of the periosteal layer, the blood effusion usually shows a dome shape leaning against the cortical bone (i.e., subperiosteal hematoma); instead, in fractures with laceration of the periosteum, the hematoma presents an irregular shape spreading to surrounding tissues (Additional file 1: Fig. 1). The periosteum presents an outer fibrous layer and an inner layer (i.e., the cambium or osteogenic layer) rich in osteoprogenitor cells [33]. In this regard, the two aforementioned pathological conditions present different healing timing. The differential diagnosis between fractures with or without periosteal disruption can be considered pivotal for the correct management of patients. Importantly, in physiological conditions, multiple collagen fibers originating from the outer layer of the periosteum penetrate through the cortical bone (i.e., the Sharpey’s fibers) guaranteeing a mechanical stabilization of the so-called periosteocortical complex [34]. For these reasons, the sonographic identification of subperiosteal hematoma can be considered an indirect sonographic sign of injury of the periosteocortical complex with disruption of the Sharpey’s fibers—i.e., a osteoperiosteal dissociation.

## Avulsion fractures

Avulsion fractures are post-traumatic cortical bone detachments at the site of tendinous or ligamentous insertion caused by a tractional force [15]. The most common anatomical locations of avulsion fractures are, respectively, the greater and lesser trochanter of the femur, the greater tuberosity of the humeral head, the medial epicondyle of the distal humerus, the superior and inferior anterior iliac spines, the tibial tuberosity, the ischial tuberosity and the base of the fifth metatarsal bone (Additional file 7: Video 6). US imaging allows clear visualization of the bony fragment and its spatial relationships with the surrounding soft tissues [15, 28]. The most common sonographic findings in avulsion fractures are (1) focal interruption of the cortical bone, (ii) local hematoma (Additional file 8: Video 7), and (3) surrounding tissue abnormalities [36]. The latter findings can be highly variable depending on the anatomical site and the dynamics of trauma—e.g., edema of the superficial soft tissues which often presents as dilatation of lymphovascular braches of subcutis [8, 9], bursitis, and articular effusion in case of intra-articular fractures [25]. Indeed, excessive tractional forces along the capsule of the joint can often lead to an avulsion fracture involving the articular surface. The effusion in case of intra-articular fracture can be composed of blood—i.e., hemarthrosis with a homogeneous sonographic pattern—or a mix of blood and bone marrow, also known as lipo-hemarthrosis. The latter pathological condition usually presents a double sonographic pattern with a hypoechoic layer of blood and a hyperechogenic fat layer—i.e., the fat-fluid sign. Importantly, in the pediatric population avulsion fracture can selectively involves the cartilaginous epiphysis resulting in a mechanical dissociation of the interface epiphysis–metaphysis (Fig. 4). This peculiar pathological condition is mainly related to the low mechanical resistance of the cartilaginous transitional plate interposed between the epiphysis and metaphysis—i.e., the physis.

Lazović et al. [41] have evaluated 243 young athletes with an anamnestic and clinically suspected apophyseal injury of the lower limb, confirming the diagnosis in 80 cases with X-ray and in 97 cases with ultrasonography. Pisacano et al. [42] have also stated that sonography should be considered an alternative imaging modality to MRI in patients in whom conventional radiography fails to reveal a clinically suspected avulsion of the pelvis.

## Stress fractures

**Fig. 5:**
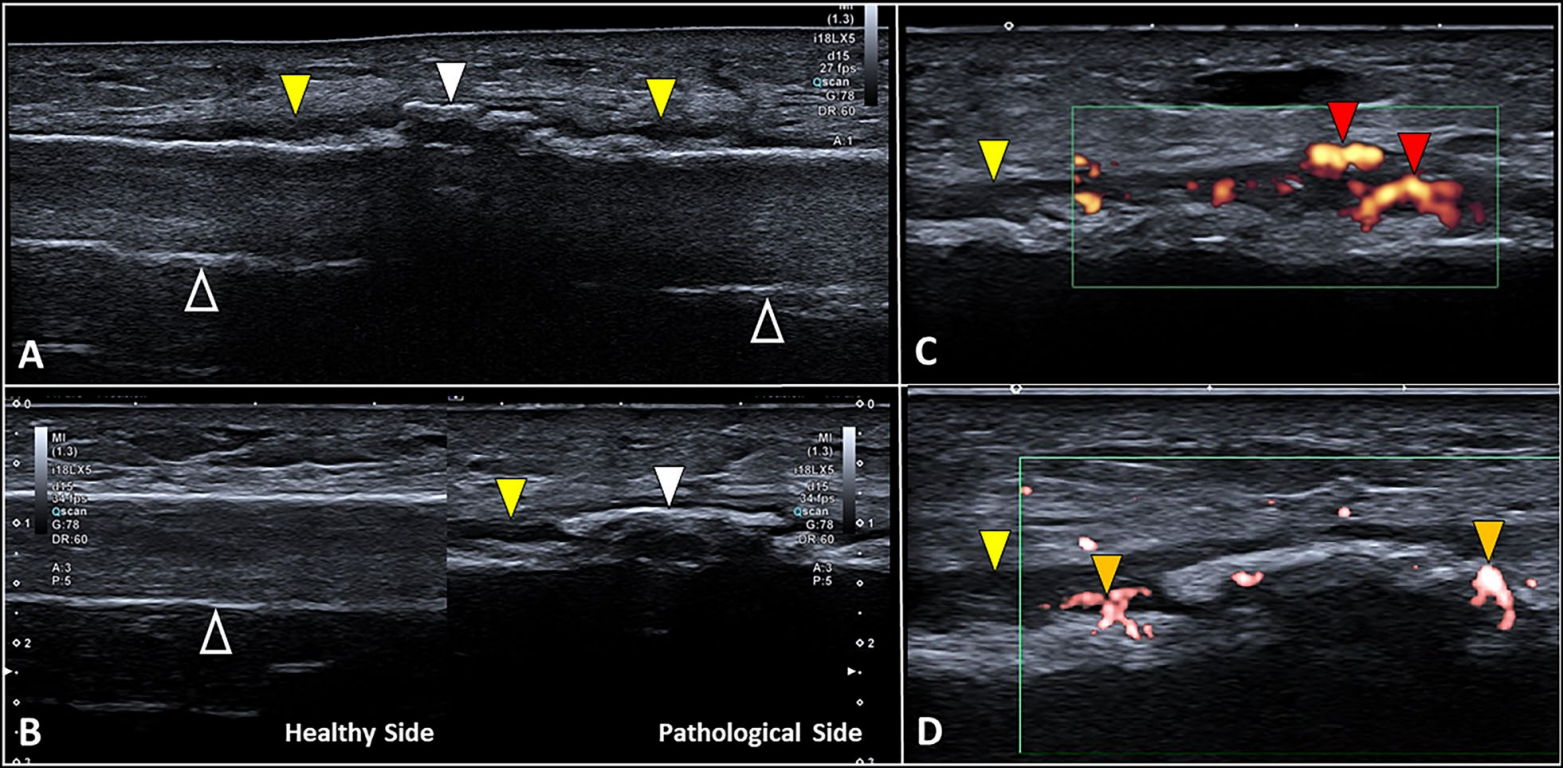
Stress fractures and healing phases. Focal thickening of the periosteum (*yellow arrowhead*), the disappearance of the reverberation artifact (*void arrowheads*) of the cortical bone, and lamellar calcifications (*white arrowhead*) within the periosteum are the most common sonographic findings in the stress fractures (**A**, **B**). Using high-sensitive color Doppler to follow up the healing phases of the stress fracture, microvasculature (*red arrowheads*) within the thickened periosteum (*yellow arrowhead*) (**C**) and penetrating vessels (*orange arrowheads*) crossing through the cortical bone (**D**) can be observed

Stress fractures are traditionally classified in fatigue fractures, related to the application of abnormal load on a healthy bone, and insufficiency, fractures due to the application of a normal load on a pathological/weakened bone [15, 28]. Stress fracture of the lower limbs is among the most frequent type of fracture in sports activities [43]. The mainly affected bones are, respectively, the tibia, the metatarsal bones, femur, fibula, pelvis, and sesamoids [44]. Instead, in older patients with osteoporosis and neurological disorders, the most affected anatomical sites are, respectively, the pelvis, the sacrum, and the femoral neck [15]. X-rays are in many cases unable to identify stress fractures, especially in the initial phase of the disease. In this way, US imaging can be considered a suitable diagnostic tool to early visualize surrounding soft tissue abnormalities before the interruption of the cortical bone develops (Table 1) [15, 28]. Of note, the aforementioned indirect sonographic signs of the “pre-fracture phase” can be easily identified in superficial bones; instead, for more deep bones—such as the pelvis or sacrum—second-level diagnostic imaging (e.g., magnetic resonance imaging) is often necessary to fully evaluate the surrounding soft tissues and the eventual bone marrow edema. Soft tissue edema can present multiple sonographic patterns related to the eventual involvement of the tiny lymphovascular plexus within the dermo-epidermal complex and/or the larger lymphovascular branches located within the fibrous scaffold of subcutaneous tissue [8, 9]. In adults, pathological thickening of the periosteum usually appears as a clearly visible hypoechoic band running just over the cortical bone [34]. The color/power Doppler assessment should be performed in each and every patient to evaluate the perfusion pattern (Fig. 5) [15, 28].

**Table 1:** Soft tissue abnormalities in stress fractures

Anatomical site	Sonographic findings
Periosteum	Hypoechoic thickening of the periosteal layer
	Periosteal delamination with multi-layered pattern
	Hypo/anechoic subperiosteal effusion *
	Hypervascularization of the periosteum (color/power Doppler)
Superficial soft tissues	Dermal edema and/or dilatation of lymphatic collectors of subcutis
	Hypervascularization of the soft tissues (color/power Doppler)

*Mechanical detachment of the periosteum from the underlying cortical bone

Banal et al. [45] have reported a sensitivity of 83% and specificity of 76% of ultrasonography in the diagnosis of metatarsal bone stress fractures evaluating 37 patients with both ultrasound imaging and MRI. Kozaci et al. [46] have demonstrated a sensitivity of 93% and a specificity of 89% in the diagnosis of low-energy fractures of metatarsal bones compared with radiography.

## Ultrasound assessment of fracture healing

Combining grayscale and color/power Doppler modalities a sonographic follow-up of the healing phases of bone fracture can be performed in clinical practice. The vascular invasion at the site of the callus and its perfusion pattern can be precisely assessed, especially accurately setting the Doppler imaging to depict small vascular elements with slow blood flows (Fig. 5) [13, 47].

In this sense, the authors propose the following basic technical tips to perform a correct assessment of the local microvasculature: (1) a large amount of gel should be used to minimize the involuntary compression of the small vessels with the ultrasound probe—i.e., the “suspension technique,” (2) the probe should be kept extremely firm during Doppler evaluation to avoid/minimize motion artefacts with fake vascular signals, (3) pulse repetition frequency should be kept as low as possible to depict small-size vascular elements and slow blood flows, (4) we suggest to increase the gain of Doppler until it reaches background noise, and then (by slowly decreasing it) is possible to remove the unreal signals, preserving the real microvascular network, (5) the size of Doppler box should be accurately evaluated considering a significant reduction in Doppler sensitivity for a too much large region of interest. Likewise, modern ultrasound equipment can also present dedicated software to optimize the visibility of small vessels with slow flows usually known as high-sensitive Doppler modalities—e.g., the superb microvascular imaging (SMI) or the microvascular flow imaging (MVFI).

Interestingly, it has been widely demonstrated that vascular signals within the fractured segment of the bone progressively reduce as the callus develops [48]. Likewise, persistent hypervascularization within the fracture site can predict a delay in the development of callus. X-rays are usually poorly accurate in the early detection of callus formation because it takes 6 to 8 weeks for callus to be seen on conventional radiographs [49]. Instead, US imaging can predict the callus development and an eventual non-union of the bone fragments can be demonstrated earlier compared to X-rays [50].

In order to correctly interpret the sonographic findings, a comprehensive knowledge of the multiple and progressive stages of the bone fracture healing process is essential (Table 2) [50]:

**Table 2:** Healing phases of bone fracture

Timing	Sonographic findings
7 days	Hypo/anechoic hematoma surrounding the fracture site
10–16 days	Hypoechoic fibrous callus within and around the fracture site
> 20 days	Hyperechoic partially-calcified callus with incomplete acoustic shadow
> 35 days	Hyperechoic calcified callus with complete acoustic shadow

*7 days: during the first week, a hematoma can be observed surrounding the fracture site and presenting as a hypoechoic or anechoic irregular area (Additional file 9: Video 8). In some cases, high-sensitive power Doppler allows to visualize the active bleeding within the pericortical hematoma (Additional file 10: Video 9).

*10–16 days: a hypoechoic, solid coat—mainly composed of fibrin matrix, fibroblasts, and collagen fibers—surrounds the fracture site defining the so-called “fibrous callus” (soft callus, primary callus). At this stage, many vascular elements originating from the periosteum (Additional file 11: Video 10) and surrounding soft tissues encircle and penetrate through the soft callus (Additional file 12: Video 11) to promote the healing processes. If the bony fragments are not correctly aligned, aberrant healing processes can lead to the development of a hypertrophic primary callus.

*> *20 days*: callus progressively calcifies and increases its echogenicity. Histologically, the fibro-vascular tissue develops firstly a cartilaginous metaplasia followed by bone metaplasia with a replacement of chondrocytes with osteocytes. The latter are highly differentiated cells producing bone trabeculae in the subperiosteal space—i.e., lamellar calcification pattern (Fig. 5). Tiny vascular elements travel within the thickened periosteum and surround the lamellar calcifications of the callus identifying a still active healing process (Additional file 13: Video 12).

**Fig. 6:**
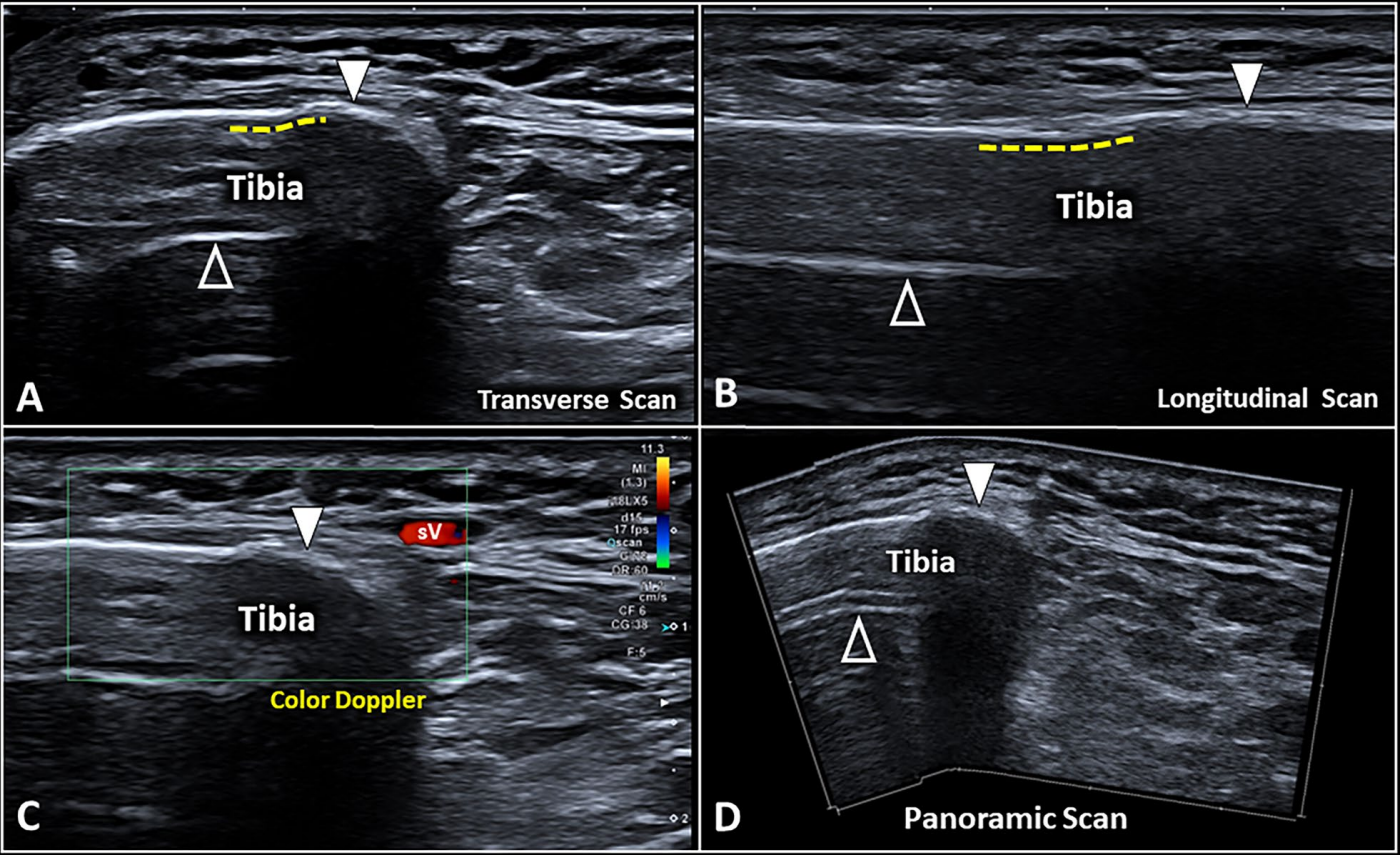
Advanced stage of the bone callus. In the advanced stage, the bone callus (*white arrowhead*) presents as a hyperechoic line similar to the surrounding normal bone cortex, but the underlying reverberation artifact (*void arrowhead*) can be absent (**A**). Of note, the aforementioned artifact stops abruptly exactly at the transitional zone (*yellow dotted line*) from the normal bone cortex to the bump of the callus (*white arrowhead*) (**A**, **B**). No vascular signals (**C**) can be visualized within/surrounding the bone callus (*white arrowhead*) defining the completed healing status of the bone fracture (**D**). *sV* superficial vein

*> *35 days*: callus is completely calcified and reflects US beam as the normal cortical bone. The irregular shape of the calcified callus can be considered a useful landmark to differentiates it from the physiological bony cortex which shows a linear pattern (Fig. 6).

US imaging allows for assessing the bone callus through all the four aforementioned phases of the healing process; instead, only the last (advanced) stage is usually visible on conventional X-rays. Each and every aforementioned phase can be assessed by combining the grayscale modality and color/power Doppler. Indeed, in the early phases, the presence of vascular signals can be considered a positive sonographic finding suggesting an active phase of healing (Additional file 11: Video 10, Additional file 12: Video 11, Additional file 13: Video 12) [51, 52]. In advanced stages (> 3 months), the persistent vascularization of the fracture site can be related to a delayed union of the bony segments [53]. In the latter case, dynamic sonographic assessment can also be performed to mechanically stress the fracture and check the eventual instability of the “immature” callus mainly composed of fibrocartilaginous tissue [15, 54]. The type of dynamic maneuver is highly variable depending on the anatomical site of fracture. We recommend firmly holding the probe over the fracture line with one hand and using the other one to passively move the target joint or apply stress forces over the fractured bone [55]. An unstable callus can progressively develop a cystic degeneration of its inner portion leading to pseudoarthrosis [54].

Lastly, among the emerging sonographic techniques, sonoelastography could be considered a potential additional tool to evaluate different phases of fracture healing. In this sense, elastography could be used as a direct and reproducible evaluation of the softness of bone callus in the initial fibrous phase, as well as of its hardness during the progressive calcification process. In the pertinent literature, Winn et al. [56] have proposed ultrasound elastography as a ready-to-use imaging modality to monitor the maturation process of the bone callus and accurately “dose the load” on the specific skeletal segment. 

## Pitfalls and limitations

As previously mentioned, normal bone irregularities can mimic a fracture at US examination and, among several examples, vascular channels and unfused bone centers are the most common examples (Fig. 2) [25]. Vascular channels, however, usually present as very localized cortical irregularities which rapidly disappear gently shifting the probe. Moreover, they are not associated with surrounding soft tissue edema and an accurate color/power Doppler assessment can demonstrate the vessels penetrating the bony cortex (Additional file 2: Video 1). Likewise, the differential diagnosis between bone fractures and painful (post-traumatic) unfused bone centers can be particularly challenging; indeed, the latter simultaneously show an interruption of the cortical bone coupled with local vascular signals like a fracture [57]. Of note, the unfused bone centers are connected to the adjacent bone by a fibrous synchondrosis and usually present rounded edges compared to the sharp profile of the bone fracture [15]. Regarding the sonographic assessment of the stress fractures, it is important to underline that pathological sonographic findings involving the periosteum and bone cortex can be very nuanced in the early phase and they can be easily missed, especially for non-expert sonographer [15]. Moreover, very small vessels with slow blood flow, movements artifacts, and incorrect interpretation due to random sampling are usually considered common pitfalls in the assessment of perfusion pattern of bone callus [1].

## Tips and tricks

We strongly suggest performing a local sono-palpation over the bone irregularity in the attempt to exactly reproduce the pain usually complained by the patient [58]. Indeed, the aforementioned simple maneuver can be useful in clinical practice to optimize the differential diagnosis between pathological bony abnormalities and normal findings [59]. On the other hand, if the target anatomical area to assess is very painful reducing the compliance of the patient to the ultrasound examination, a large amount of gel (the “suspension technique”) can be used to avoid physical contact between the ultrasound probe and the skin. Likewise, in the presence of doubtful/ambiguous sonographic findings, the comparative ultrasound examination [59, 60] of the healthy vs. painful side can be considered an essential phase of the assessment.

## Conclusions

US is an emerging valuable diagnostic tool for bone fractures due to its wide availability and thorough bone assessment both in emergency and follow-up [23]. Medical history, physical examination, and knowledge of US bone fracture patterns may allow to reach an early diagnosis thus reducing the use of more expensive or radiation-based methods. The integration with other imaging methods should be always considered in doubtful cases or for surgical planning.

## Supplementary Information

**Additional file 1: Figure S1.** Hematoma and Periosteum. A bone fracture can present histological preservation of the periosteum (yellow) with a dome shape hematoma (red) **(A)** or can show a post-traumatic disruption of the periosteal layer (yellow) with a diffusion of the blood (red) within the surrounding soft tissues **(B)**. The subperiosteal hematoma (red) is often related to a mechanical disruption of the Sharpey’s fibers (black lines) with dissociation of the anatomical interface between the cortical bone and periosteum (yellow) **(C)**.

**Additional file 2: Video 1.** High-sensitive power Doppler clearly shows feeding vessels penetrating the bone cortex and traveling within nutritional foramina.

**Additional file 3: Video 2.** Cartilaginous plate in between the metaphysis and epiphysis of the distal femur in young volunteer.

**Additional file 4: Video 3.** Long-axis view of a post-traumatic rib fracture with bulging of the periosteum and subperiosteal hematoma.

**Additional file 5: Video 4.** Sonographic tracking of a post-traumatic rib fracture. Of note, the transverse acoustic window easily shows the focal interruption of the cortical bone.

**Additional file 6: Video 5.** Hill–Sachs defect presents as a depression of the cortical bone in the posterolateral side of the humeral head.

**Additional file 7: Video 6.** A longitudinal scan shows the avulsion fracture of the base of the 5th metatarsal bone at the attachment site of the lateral cord of plantar fascia after an ankle sprain.

**Additional file 8: Video 7.** A transverse scan shows the hematoma surrounding the bony fragment detached from the base of the 5th metatarsal bone after an ankle sprain.

**Additional file 9: Video 8.** Local hematoma can be nicely observed surrounding the cortical irregularity due to a post-traumatic fracture of the distal radius.

**Additional file 10: Video 9.** High-sensitive power Doppler allows to observe the microvasculature within the hematoma surrounding the bone fracture.

**Additional file 11: Video 10.** High-sensitive power Doppler shows the vascular invasion—originating from the thickened periosteum—of the fibrous callus during the healing phase.

**Additional file 12: Video 11.** The color Doppler clearly shows neovessels located within the superficial portion of the (hypoechoic) fibrous callus in a patient with a post-traumatic fracture of the humeral neck.

**Additional file 13: Video 12.** High-sensitive power Doppler shows the microvasculature within the thickened periosteum and around the partially calcified callus.

## Data Availability

Not applicable.

## Abbreviations

CT: Computed tomography
MRI: Magnetic resonance imaging
US: Ultrasound
XR: X-ray
