# Supplementary material for: Ultrasound imaging of bone fractures
Source: Insights Imaging. 2022 Dec 13;13:189. doi: 10.1186/s13244-022-01335-z (PMC9748005; doi:10.1186/s13244-022-01335-z)
Supplement: Supplementary file 1 — Additional file 1: Figure S1. Hematoma and Periosteum. A bone fracture can present histological preservation of the periosteum (yellow) with a dome shape hematoma (red) (A) or can show a post-traumatic disruption of the periosteal layer (yellow) with a diffusion of the blood (red) within the surrounding soft tissues (B). The subperiosteal hematoma (red) is often related to a mechanical disruption of the Sharpey’s fibers (black lines) with dissociation of the anatomical interface between the cortical bone and periosteum (yellow) (C). [file 13244_2022_1335_MOESM1_ESM.pdf]

ELECTRONIC SUPPLEMENTARY MATERIAL

Ultrasound Imaging of Bone Fractures

Supplementary Figure 1

Hematoma and Periosteum.

A bone fracture can present histological preservation of the periosteum *(yellow)* with a dome shape hematoma *(red)* **(A)**; or, can show a post-traumatic disruption of the periosteal layer *(yellow)* with a diffusion of the blood *(red)* within the surrounding soft tissues **(B)**. The subperiosteal hematoma *(red)* is often related to a mechanical disruption of the Sharpey's fibers *(black lines)* with dissociation of the anatomical interface between the cortical bone and periosteum *(yellow)* **(C)**.

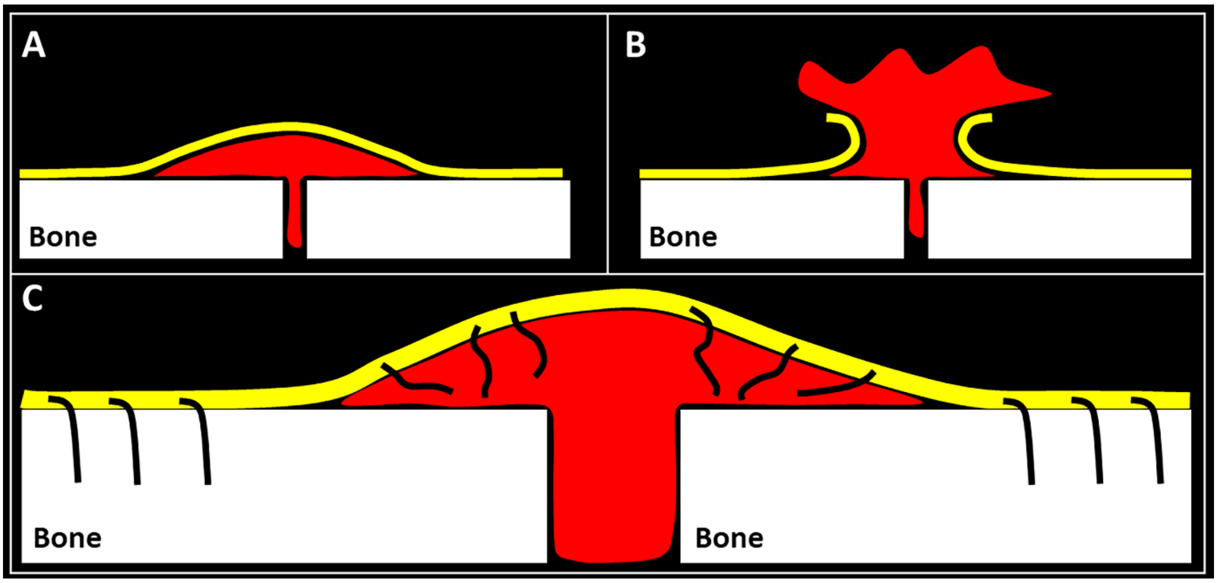

Video legends

Video 1

High-sensitive power Doppler clearly shows feeding vessels penetrating the bone cortex and, traveling within nutritional foramina.

Video 2

Cartilaginous plate in between the metaphysis and epiphysis of the distal femur in young volunteer.

Video 3

Long-axis view of a post-traumatic rib fracture with bulging of the periosteum and subperiosteal hematoma.

Video 4

Sonographic tracking of a post-traumatic rib fracture. Of note, the transverse acoustic window easily shows the focal interruption of the cortical bone.

Video 5

Hill-Sachs defect presents as a depression of the cortical bone in the posterolateral side of the humeral head.

Video 6

A longitudinal scan shows the avulsion fracture of the base of the 5th metatarsal bone at the attachment site of the lateral cord of plantar fascia after an ankle sprain.

Video 7

A transverse scan shows the hematoma surrounding the bony fragment detached from the base of the 5th metatarsal bone after an ankle sprain.

Video 8

Local hematoma can be nicely observed surrounding the cortical irregularity due to a post-traumatic fracture of the distal radius.

Video 9

High-sensitive power Doppler allows to observe the microvasculature within the hematoma surrounding the bone fracture.

Video 10

High-sensitive power Doppler shows the vascular invasion - originating from the thickened periosteum - of the fibrous callus during the healing phase.

Video 11

The color Doppler clearly shows neovessels located within the superficial portion of the (hypoechoic) fibrous callus in a patient with a post-traumatic fracture of the humeral neck.

Video 12

High-sensitive power Doppler shows the microvasculature within the thickened periosteum and around the partially calcified callus.
